# Climate Change Hastens the Conservation Urgency of an Endangered Ungulate

**DOI:** 10.1371/journal.pone.0022873

**Published:** 2011-08-03

**Authors:** Junhua Hu, Zhigang Jiang

**Affiliations:** 1 Key Laboratory of Animal Ecology and Conservation Biology, Institute of Zoology, Chinese Academy of Sciences, Beijing, China; 2 Chengdu Institute of Biology, Chinese Academy of Sciences, Chengdu, Sichuan, China; Smithsonian's National Zoological Park, United States of America

## Abstract

Global climate change appears to be one of the main threats to biodiversity in the near future and is already affecting the distribution of many species. Currently threatened species are a special concern while the extent to which they are sensitive to climate change remains uncertain. Przewalski's gazelle (*Procapra przewalskii*) is classified as endangered and a conservation focus on the Qinghai-Tibetan Plateau. Using measures of species range shift, we explored how the distribution of Przewalski's gazelle may be impacted by projected climate change based on a maximum entropy approach. We also evaluated the uncertainty in the projections of the risks arising from climate change. Modeling predicted the Przewalski's gazelle would be sensitive to future climate change. As the time horizon increased, the strength of effects from climate change increased. Even assuming unlimited dispersal capacity of gazelles, a moderate decrease to complete loss of range was projected by 2080 under different thresholds for transforming the probability prediction to presence/absence data. Current localities of gazelles will undergo a decrease in their occurrence probability. Projections of the impacts of climate change were significantly affected by thresholds and general circulation models. This study suggests climate change clearly poses a severe threat and increases the extinction risk to Przewalski's gazelle. Our findings 1) confirm that endangered endemic species is highly vulnerable to climate change and 2) highlight the fact that forecasting impacts of climate change needs an assessment of the uncertainty. It is extremely important that conservation strategies consider the predicted geographical shifts and be planned with full knowledge of the reliability of projected impacts of climate change.

## Introduction

In recent decades, global climate has undergone dramatic changes, which are expected to continue into the 21^st^ century [1]. It is increasingly clear that rapid climate change is profoundly affecting the Earth's biodiversity [2], [3] and the challenges to conservation and environment management in the face of these changes are immense [4]–[6]. For example, anthropogenic climate change is already affecting the physiology, phenology, reproductive output, survival rate and distribution of many species [2], [7]–[10]. Evidence is accumulating that imminent changes to the global climate will potentially result in high extinction rates around the world [11], [12].

There is a growing consensus that biodiversity conservation must take the impacts of climate change into consideration [3], [6], [13]. Endemic species, because of their small geographic range, are likely to be more dispersal-limited and less able to adapt to a rapidly shifting climate than other species [14]. Although the influence of current climate on the distribution of endemic species is unclear, richness of endemic species is more strongly related to factors directly affecting long-term survival and speciation than current climate [15], [16]. Nevertheless, Ohlemüller *et al.* [14] found areas with high numbers of small-range species to be colder and located at higher elevations than surrounding regions, suggesting that these are interglacial relict areas for cold-adapted species with a high vulnerability to future global warming. Species can respond to climate change by shifting distribution to follow changing environments or by adapting to altering conditions. If unable disperse or adapt, species can remain in isolated pockets of unchanged environment (“refugia”) or, more likely, will become extinct [11], [13], [17]. Although some attention has been given to the last three options [4], [18], [19], using “species distribution models” (SDMs) to project how the distributions of species may change under different scenarios of future climate change has become especially popular [5], [6], [20]. In this regard, rapid progress in predicting the distributions of species has been made and tools are now available to assess the impacts of climate change on species [11], [21]–[23].

Despite their popularity, it is widely acknowledged that SDMs over-simplify the processes governing the geographic distributions of species [23], [24]. In fact, of the many ecological and evolutionary processes which are expected to determine contemporary distributions of most species [25], [26], several are poorly accounted for when applying SDMs [23]. In addition to ecological uncertainties, recently much attention has been paid to address other uncertainties embedded in SDMs [27]–[29]. The sources of uncertainty are diverse and may arise because of differences in data sources and statistical methods used in SDMs (e.g. measurement errors, small sample size, missing covariates and biased samples) [28], [30]. For example, a large number of general circulation models (GCMs) have been developed simultaneously by different meteorological research centers to represent physical processes in the atmosphere, ocean, cryosphere, and land surface. Concurrently, four scenarios have been defined. Each is an alternative image of how the future might unfold assuming a certain level of future greenhouse gas emissions [1]. Regardless, SDMs provide a useful way to incorporate future conditions into conservation and management practices and decisions when the uncertainties of model projections with the risks of taking the wrong actions or the costs of inaction are balanced [3], [26], [28].

Przewalski's gazelle (*Procapra przewalskii*) is one of the most endangered ungulates in the world [31], [32]. It is endemic to China and a conservation focus on the Qinghai-Tibetan Plateau [33]. The species was once distributed throughout Qinghai, Ningxia, Inner Mongolia and Gansu Provinces, China. Increasing human activity over the last century has resulted in continuing habitat destruction and range reduction for the gazelle [34]. The gazelle was listed as critically endangered by the IUCN until 2008 when it was reclassified as endangered [35], based on newly discovered populations with approximately 300 gazelles in Tianjun County (locating in the northwest of Qinghai Lake watershed area), and in Wayu and Ranquhu (the southwest of Qinghai Lake) [36]. Today only several hundred individuals survive in isolated localities around Qinghai Lake [33], [37].

Key assumptions in SDMs are that species are at equilibrium with the environments, and that relevant environmental gradients have been adequately sampled [38]. In this respect, although Przewalski's gazelle is not in climatic equilibrium, and its restricted distribution is the product of human activities [33], [36], the presence of gazelles (recorded during prolonged surveys) are thought to be at equilibrium with the socio-ecological environments [23], [36].

To explore how Przewalski's gazelle may be impacted by currently projected climate change, we used SDMs to model the suitable habitats under different climate change scenarios for 2020, 2050 and 2080 and assessed the uncertainty in the projections. In particular we asked: will projected climate change alter current suitable habitat of the gazelle? Will there be more suitable habitat or less? To what extent will the gazelle be threatened by climate change in the future? Our study will inform relevant policy makers and conservation authorities of the potential vulnerabilities of this endangered ungulate to climate change, and will guide future conservation planning not only for the gazelle, but for other threatened ungulate species.

## Methods

### Species occurrence data

The study region here encompassed the historical and current ranges of Przewalski's gazelle. Investigations for the species were conducted for historical ranges in Inner Mongolia, Gansu, Xinjiang and Qinghai Provinces, China [31], [33]. For current distribution ranges, a long-term and regular monitoring program of known populations was implemented mainly using transect census [33], [36], [39], [40]. We collected species occurrence data based on extensive field surveys for gazelles during the period of 2002–2008. Historical distribution was determined through literature records [33]. All occurrence data were lumped together and treated the same with a total of 3897 presence records and no absence data. Due to the clustering of initial records, 117 presence point data (Table S1) were retained for further analysis in Maxent after duplicates in the same 1×1 km grid cells were removed using ArcGIS 9.2 (ESRI, Redland, USA).

### Environmental predictors

We used 19 environmental predictors across four types of data: (1) *Climate*-12 predictors, i.e. annual mean temperature, mean diurnal range, isothermality, temperature seasonality, maximum temperature of the warmest month, mean temperature of the wettest quarter, mean temperature of the warmest quarter, annual precipitation, precipitation of the wettest month, precipitation of the driest month, precipitation of the wettest quarter and precipitation of the warmest quarter from WorldClim 1.4 [41]. (2) *Habitats*- land cover layer [42] and the normalized difference vegetation index (NDVI) for April, May, July and August, respectively (http://www.data.ac.cn/index.asp). (3) *Human impact*-human influence index (HII, an estimate of human influence based on human settlement, land transformation, accessibility and infrastructure data) [43]. (4) *Topography*-elevation from the Hydro1K dataset [44]. All predictor layers were at a resolution of 1×1 km to match the presence data. The 19 predictors were considered important based on the outputs of jackknife analyses among the raw 38 variables (Figure S1). The model conducted with these predictors performed well and outperformed the model conducted using a set of uncorrelated (*r*<0.8) predictors [45].

### Climate change scenarios

For climate change scenarios we referred to the Intergovernmental Panel on Climate Change (IPCC) [1] Special Report on Emissions Scenarios, which describes the relationships between the forces driving greenhouse gas and aerosol emissions and their evolution during the 21^st^ century. Each scenario represents different assumptions regarding demographic, social, economic, technological, and environmental developments that diverge in increasingly irreversible ways. We selected two greenhouse gas emission scenarios (GESs; A2a and B2a) to assess plausible futures based on a range in human choices over the next few decades. The A2a scenario describes a highly heterogeneous future world with regionally oriented economies. The main driving forces are a high rate of population growth, increased energy use, land-use changes and slow technological change. The B2a scenario is locally and regionally oriented but with a general evolution towards environmental protection and social equity. Compared to B2a, A2a projects a higher rate of population growth, a larger increase in GDP and faster land-use changes, but less diverse technological changes. B2a projects resource conservation efforts beginning in the early decades of this century and CO_2_ emissions declining by midcentury [1]. Given the great uncertainty in predicting future climate, we used projections from three internationally recognized GCMs, i.e. CCCMA (Canadian Centre for Climate Modeling and Analysis) [46], CSIRO (Commonwealth Scientific and Industrial Research Organization) [47] and HADCM3 (Hadley Centre Coupled Model version 3) [48], that simulated the impact of the A2a and B2a scenarios on future climate conditions. These are considered the most advanced simulations of global climate system responses to increasing greenhouse gas concentrations currently available.

In order to explore the potential range of Przewalski's gazelle in the future we extracted the above climate predictors across the three GCMs under the two GESs for the years 2020, 2050 and 2080. Estimations of future non-climatic predictors were not available because a wide range of socio-economic drivers would affect those factors. The extrapolation of past trends in non-climatic variables to the future was considered conservative estimators of the future in order to avoid misleading conclusion due to over-simplifications [5], [20].

### Niche-based models

**Figure 1:**
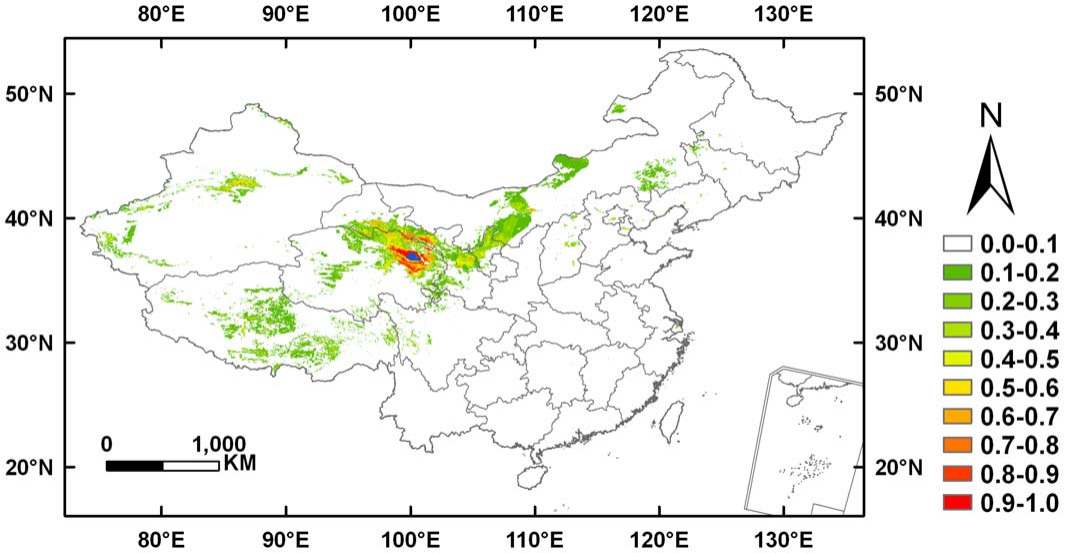
Predicted probability distribution for *Procapra przewalskii* using Maxent at 1×1 km. The lapis lazuli area indicates the Qinghai Lake.

We implemented Maxent [49] (version 3.3.1; www.cs.princeton.edu/~schapire/maxent/) to model the suitability of habitat for Przewalski's gazelle (Fig. 1) [45]. Maxent, a machine learning method, is one of the most popular SDMs and is among the best-performing modeling approaches using presence-only data [49]–[51]. It satisfies a set of constraints representing the incomplete information on the distribution and, subject to those constraints, finds the probability distribution using the maximum entropy principle [49]. We adhered to the default settings for the regularization multiplier (1), maximum number of iterations (500), convergence threshold (10^−5^) and maximum number of background points (10 000). We generated models randomly assigning 80% of occurrences as training data with the remaining 20% used as test data. We ran five cross-validate replicates for each model. Selection of “features” (predictors) was carried out automatically, following the default rules dependent on the number of presence records. We used the easily interpretable logistic output format conditioned on the environmental variables in each grid cell [50] with suitability values ranging from 0 (unsuitable habitat) to 1 (optimal habitat).

We projected the current prediction [45] on the future climate scenarios and produced 90 future distribution models [ = 5 models×18 projections ( = 3 GCMs×2 GESs×3 time slices)]. Growing concerns have emerged that excessive variability is introduced when applying ensemble-forecasting approaches which fit a number of alternative models (i.e. the use of multiple models) to reach a consensus scenario, thus possibly compromising policy decisions [52]. The basis of the consensus approach is that different predictions are copies of possible states of the real distributions, and they form an ensemble. Because different SDMs provide considerably variable performance [53]–[55] and contribute to the largest variation in the projections of impacts of climate change [28], we addressed variability concerns by using the cross-validate replicates from Maxent as proxies for different single-models in consensus methods [52]. We employed the consensus method, namely Mean (based on mean function), which forms a representation of the most commonly used techniques and has been shown to yield robust predictions [55].

We used a suitability threshold to derive projected presence-absence distributions from the logistic outputs. As the choice of a threshold has a great effect on the projected map but there is still no consensus on the selection of optimal threshold [45], [56], three different thresholds were implemented. Because the threshold indicating maximum training sensitivity plus specificity is considered as a more robust approach [53], [56], we used it to conduct the conversion into presence-absence predictions. To evaluate the degree of climate change risk, as an alternative approach, we also used two fixed thresholds of 0.8 and 0.95 [57].

### Spatial index for potential impacts of climate change

We used three approaches to assess the impacts of climate change on the potential habitat ranges. First, range shift was calculated under two spread assumptions: null spread (no spread ability of gazelles) and full spread (unlimited ability to spread). Under the assumption of null spread, only the overlap habitat between current and future ranges was considered suitable for gazelles. Under the full spread assumption, the gazelle populations could reach all new potential habitat ranges. To assess range variation at the pixel level, we summed the potential range loss (RL) by pixel and related this to the predicted current range (CR) by pixel. Under the full spread, the percentage of range gained (RG) by pixel was assessed by the same procedure; we estimated the percentage of predicted range change (C) by pixel [5] using
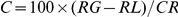
and turnover (T) by pixel using
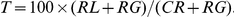



Second, we conducted a comparison with a formula that uncovers the maximal divergence among time slices:
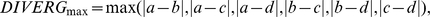
where 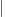 is the absolute value of the difference between two time slices; max operator is the maximum difference among time slices; *a*, *b*, *c*, and *d* represent the current, 2020, 2050 and 2080 models, respectively [5].

Third, based on the predicted distributions and using spatial analysis tools in ArcGIS (ESRI, Redland, USA), we extracted the probability of occupancy for known localities (i.e. presence records) of gazelles for the four time slices considered (current, 2020, 2050 and 2080). We then characterized the trends in the projected probability of occurrences [58].

### Extinction risk

In line with IUCN Red List criterion A3(c), based on the predicted reduction in range size in the future, we assigned the gazelle to a threat category. The threat categories and their thresholds are as follows [59]: extinct, species with a projected range reduction of 100% in the future; critically endangered, projected range reduction of >80%; endangered, projected range reduction of >50%; and vulnerable, projected range reduction of >30%. Although it is important to note that the Red Listing approach is simplistic and general and considers only the effects of projected climate change, it provides a synthetic overview of species-specific threats due to climate change [20]. We estimated the extinction risk under assumptions of: 1) null spread, where range reduction was calculated as the percentage of RL, and 2) full spread, where range reduction was calculated as C.

### Uncertainty analysis

We used Kolmogorov-Smirnov tests to check normality of data and transformed data to meet assumptions of normality and homogeneity of variances. Multivariate analysis of variance (MANOVA), which takes into account collinearity among response variables, was performed to test the effects of time slice, threshold and GCMs, and their interactions on the four variables (i.e. the percentage of range lost, gain, change, and turnover) for estimating the impacts of climate change. A significant MANOVA was followed up with univariate ANOVAs. All data of the percentage of range gain were logarithmic transformed (*log_10_*) prior to analyses, and the data of the percentage of range change were abs and *log_10_* transformed. These statistical analyses were performed using SPSS 15 software (SPSS Inc., Chicago, USA).

## Results

### Potential impacts of climate change

**Figure 2:**
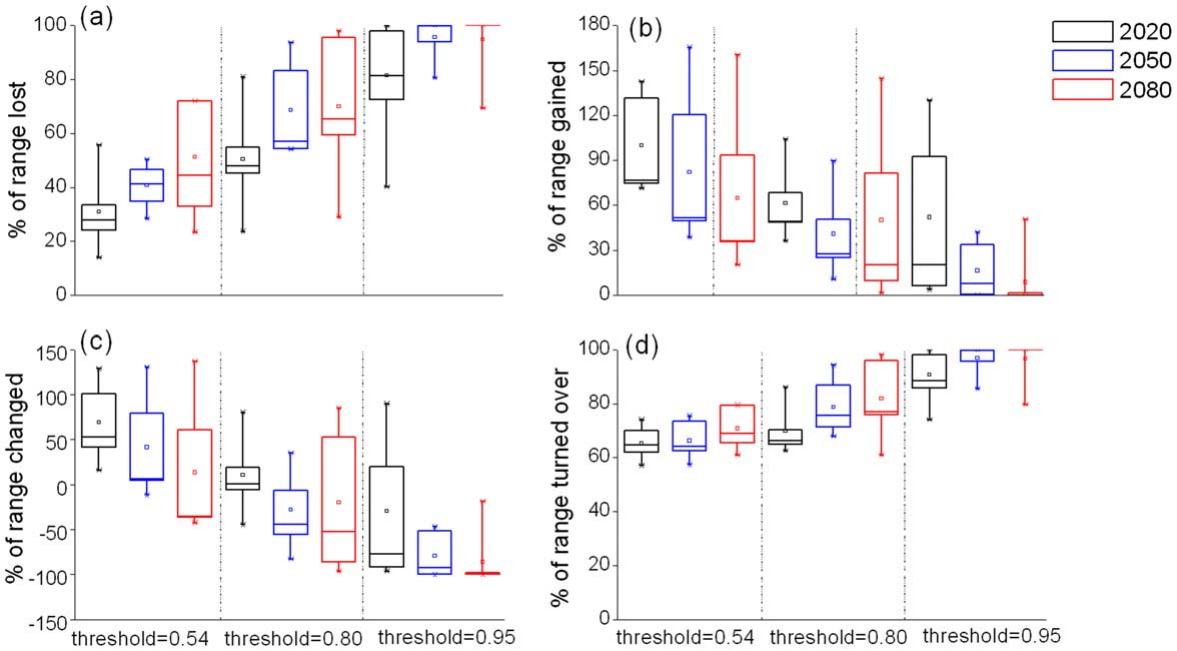
Projected impacts of climate change on the distribution of *Procapra przewalskii*. Percentage of range loss, range gain, range change and range turnover as predicted using presence by pixel across the three general circulation models (CCCMA, CSIRO and HADCM3) and the two climate change scenarios: A2a (liberal) and B2a (conservative), for three time slices (2020, 2050 and 2080). The solid horizontal line represents the median, the square symbol represents the mean, edges of box are quartiles, whiskers are 5th and 95th percentiles and circles are outliers.

The potential range of Przewalski's gazelle was discernibly impacted by projected climate change (Fig. 2). Across the GCMs and GESs, it was clear that the strength of the impacts increased as the time horizon or the cut-off value increased. With the threshold indicating sensitivity-specificity sum maximization, for the years 2020, 2050 and 2080, the average percentage of range loss was 31%, 41% and 51% respectively. Under the full spread assumption, the average percentage of range gain for the same years was 100%, 82% and 65%. This predicted a strong turnover in range for all future time slices. Strong to small range increases were projected from 2020 to 2080.

With the threshold of 0.80, for the years 2020, 2050 and 2080, the average percentage of range loss was 50%, 69% and 70%, respectively. Under the full spread, the average percentage of range gain for the same years was 61%, 41% and 50%. A strong turnover in range was provided by all three time slices. Small range increase was projected by 2020 but the gazelle was predicted to experience a moderate reduction in suitable habitat by 2050 and 2080 (Fig. 2).

With the threshold of 0.95, for the years 2020, 2050 and 2080, the average percentage of range loss was 82%, 95% and 95%, respectively. Under the full spread, the average percentage of range gain for the same years was 52%, 16% and 9%. This gave an extremely strong turnover in range by all three time slices. Moderate to strong range reductions were projected by 2020 and later years (Fig. 2).

**Figure 3:**
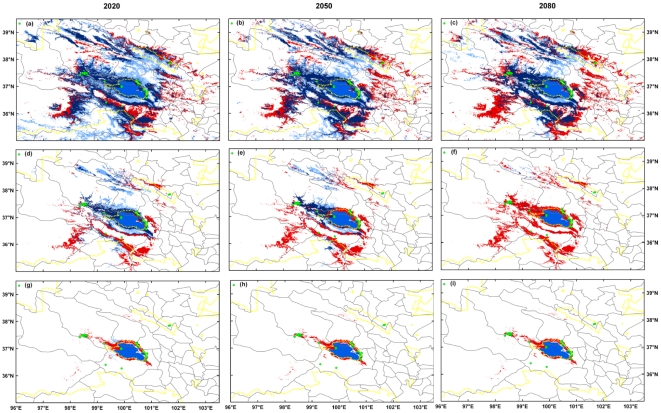
Close-up of predicted distribution of *Procapra przewalskii* for three time slices: 2020, 2050 and 2080. Models are obtained with an ensemble-forecast approach across the three general circulation models (CCCMA, CSIRO and HADCM3) and the two climate change scenarios (A2a and B2a). Suitable ranges are selected by the thresholds of 0.54, 0.80 and 0.95 (panels a–c; d–f; g–i, respectively) for current and future predictions. For all panels, red indicates the current suitable habitats predicted to be unsuitable in the future; yogo blue indicates the current nonsuitable habitats predicted to be suitable in the future and blue indicates current suitable habitats predicted to stay suitable in the future. The gray solid lines represent county boundaries and yellow dotted lines, the boundaries of protected areas. The lapis lazuli area indicates the Qinghai Lake.

Spatial outputs of the ensemble-forecast approach revealed that the potential range will be vulnerable to large variation under projected climate scenarios (Fig. 3). Under the full spread, with the threshold indicating sensitivity-specificity sum maximization, the gazelle could experience an increase in suitable habitat with more range gain than loss by 2020; the variation of range size was little with approximately equal area of loss and gain by 2050; while a considerable negative impact was suggested by 2080 with range loss more than doubling range gain (Fig. 3a–c). With the threshold of 0.80, a reduction was projected in suitable habitat for the three time slices. As the time horizon increased this negative impact was predicted to expand. For the years 2020, 2050 and 2080, the area ratio (range loss: range gain) was 1.5, 3.5 and 39.9 respectively (Fig. 3d–f). With a more restrictive threshold of 0.95, by 2020 the current suitable habitat (*c.* 5630 km^2^) was predicted to diminish to only 20 km^2^, while range gains of approximately 490 km^2^ were expected in the west and northwest of Qinghai Lake. This reduction in range size could be extremely severe with only 1 km^2^ suitable habitat by 2050 and no suitable habitat by 2080 (Fig. 3g–i).

**Figure 4:**
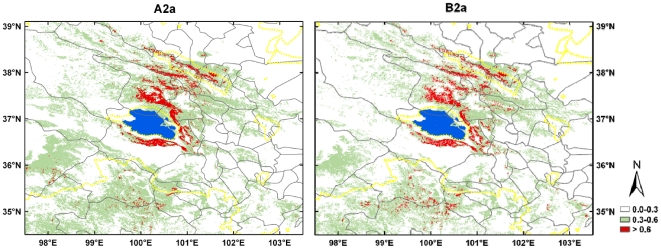
Close-up of model disagreement among time slices (current, 2020, 2050 and 2080). The disagreement is estimated through maximal divergences (DIVERG_max_) for the two climate change scenarios: A2a (panel a) and B2a (panel b). The gray solid lines represent county boundaries and yellow dotted lines, the boundaries of protected areas. The lapis lazuli area indicates the Qinghai Lake.

Spatially explicit comparisons between current and future potential ranges identified high divergences in certain areas and consistently highlighted similar maximal divergences between the scenarios A2a and B2a (Fig. 4). DIVERG_max_ predicted reductions in suitable habitat under future climate change scenarios in two regions of high probability of occupancy, situated in the south, and from east to north of Qinghai Lake.

**Figure 5:**
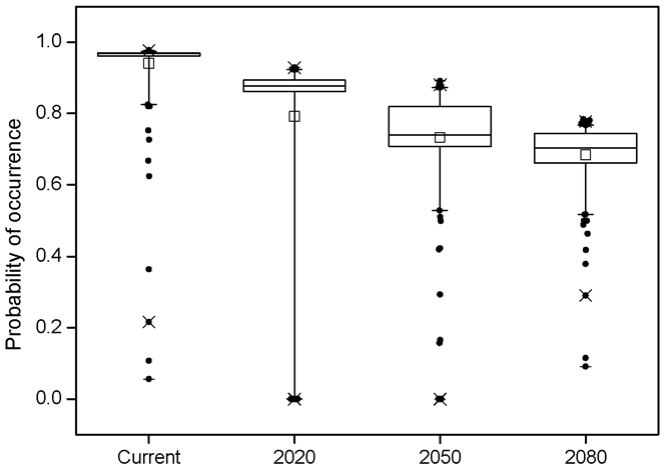
Predicted probability of occurrence for known *Procapra przewalskii* localities. The probability of occurrence is extracted for the four time slices considered (current, 2020, 2050 and 2080) based on the predicted distributions from an ensemble-forecast approach. The solid horizontal line represents the median, the square symbol represents the mean, edges of box are quartiles, whiskers are 5th and 95th percentiles and circles are outliers.

Currently known localities of gazelles were forecasted to undergo a decrease in the probability of occurrence over time (Fig. 5). Probability of occurrence was predicted to drop from 0.940 based on current data to 0.792 by 2020, 0.732 by 2050 and 0.684 by 2080.

### Extinction risk evaluation

The application of IUCN Red List A3(c) criterion highlighted that the gazelle could be severely threatened by projected climate change (Fig. 2 & 3). This also revealed the uncertainty provided by the crude spread assumptions. Based on the ensemble-forecast approach across climate scenarios, under the assumption of no spread, the gazelle would be classified as vulnerable after 2020 and became endangered for 2080 with the threshold indicating sensitivity-specificity sum maximization, while it would be endangered after 2020 and became critically endangered for 2080 with the threshold of 0.80. With the most rigorous threshold of 0.95, the species may become critically endangered by 2020 (>95% range loss), and committed to extinction after 2050. Under the full spread assumption, the results were, as expected, less severe but not optimistic. Although the species was classified as low risk across time slices with the threshold indicating sensitivity-specificity sum maximization, with the threshold of 0.80 it was classified as endangered and critically endangered by 2050 and 2080, respectively. Additionally, the gazelle would be endangered after 2020 and committed to extinction by 2080 with the threshold of 0.95.

### Relative contribution of uncertainty to projections

Based on the MANOVA, the assessment of impacts of projected climate change was significantly affected by the threshold and GCMs used. Range variation did not vary significantly with time slice. There was also no significant effect from the interactions between these three components (Table 1). On the other hand, the univariate ANOVAs revealed significant changes in range loss, range change and range turnover with time slice, but no significant change in range gain. These four measures for estimating impacts of climate change were significantly affected by both threshold and GCMs. However, no significant effects of the interactions between the three components were found.

**Table 1:** Results of both multivariate analysis of variance (MANOVA) and univariate ANOVAs on the effects of time slice, threshold, GCMs, and their interactions on the four measures for estimating potential impacts of climate change: the percentage of range loss, range gain, range change and range turnover predicted.

		Wilks' λ	*df*	*F*	*P*
Multivariate					
Time slice		0.632	6, 50	2.16	0.063
Threshold		0.161	6, 50	12.42	0.000**
GCMs		0.457	6, 50	3.99	0.002*
Time slice * Threshold		0.606	12, 66	1.15	0.336
Time slice * GCMs		0.687	12, 66	0.84	0.606
Threshold * GCMs		0.550	12, 66	1.40	0.187
Time slice * Threshold * GCMs		0.614	24, 73	0.56	0.945
Univariate					
Time slice	Range loss		2	6.219	0.006*
	Range gain		2	2.969	0.068
	Range change		2	4.169	0.026*
	Range turnover		2	3.896	0.033*
Threshold	Range loss		2	44.040	0.000**
	Range gain		2	9.325	0.001**
	Range change		2	18.316	0.000**
	Range turnover		2	47.790	0.000**
GCMs	Range loss		2	8.030	0.002*
	Range gain		2	4.980	0.014*
	Range change		2	6.046	0.007*
	Range turnover		2	4.447	0.021*
Time slice * Threshold	Range loss		4	0.264	0.898
	Range gain		4	0.358	0.836
	Range change		4	0.238	0.914
	Range turnover		4	0.483	0.748
Time slice * GCMs	Range loss		4	1.373	0.270
	Range gain		4	0.496	0.739
	Range change		4	0.684	0.609
	Range turnover		4	1.341	0.280
Threshold * GCMs	Range loss		4	0.590	0.673
	Range gain		4	0.618	0.654
	Range change		4	0.412	0.798
	Range turnover		4	1.372	0.270
Time slice * Threshold * GCMs	Range loss		8	0.308	0.956
	Range gain		8	0.438	0.888
	Range change		8	0.390	0.916
	Range turnover		8	0.267	0.971

## Discussion

### Sensitivity to climate change

Predicting the effects of anthropogenic climate change on the distributions of species is critical [3], since these changes may lead to massive species extinctions [4], [11], [20]. While some species are likely to benefit from the changes with extending ranges into currently unoccupied areas, many mammals exhibit generally predictable responses to changing climate which may alter their distribution ranges or accelerate extinction rates [6], [8], [13], [17], [20].

As expected, the projected distribution of Przewalski's gazelle resulting from several climate change scenarios suggests this species will become much more limited in suitable habitat. While the gazelle appears to gain range under the universal spread assumption, the probability of occurrence is relatively low for most new habitats. Additionally, large proportions of the current habitat of high occurrence probability are expected to become unsuitable with climate change in the future (Fig. 2 & 3).

Furthermore, the rangelands on the Qinghai-Tibet Plateau where all current Przewalski's gazelle populations now exist [33] may also be vulnerable to climate change [60], [61]. Climatic warming affects vegetation production and quality negatively [60] and important plant groups for ecosystem services may undergo species loss due to consumption by livestock with climate warming [61]. Given the competition between gazelles and domestic sheep for food as well as the habitat space conflict between gazelles and local people [33], [62], climate change will threaten the survival of gazelles.

### Threshold selection, extinction risk and the uncertainty

It is a prerequisite to predict how species will respond to anticipated climate changes in order to effectively conserve populations and reduce extinction rates. However, uncertainty surrounding the degree to which climate change will impact species presents a challenge for environmental management and policy [27], [28], [63]. It would be wise to recognize and quantify this uncertainty when developing conservation strategies [55], [64]. The choice of an optimal suitability threshold is crucial in conservation practice [56], [57]. No general-purpose rule exists for selection of an optimal suitability threshold despite many approaches available for threshold-determining (subjective or objective) [56] and this remains to be further explored in Maxent [45], [49]. Our analyses revealed that the selected threshold significantly affects the assessment of impacts of climate change on future populations of Przewalski's gazelle. The threshold indicating sensitivity-specificity sum maximization here (0.54) was lenient and produced optimistic results when evaluating the extinction risk. Future populations of the gazelle would be vulnerable or endangered under the null spread assumption but low risk under the full spread assumption. In view of the fact that gazelle populations are greatly disturbed by human activities [36], [65], [66] and the uncertainties in the simulations of climate change impacts [28], [29], [55], we should consider the more severe prediction. With more stringent strategies (the threshold of 0.8 and 0.95 here), the reduction in range size was projected for most climate change scenarios under both the null and full spread assumptions. Specifically, under the threshold of 0.95 the gazelle would have no suitable habitat by 2080 and subsequently become critically endangered or extinct.

Several studies have extrapolated to alarming extinction risks in the future [11], [20], despite criticisms that the use of IUCN Red List Criteria [59] for estimating this risk is too loose [67]. While acknowledging the uncertainty from SDMs and GCMs [28], we addressed how sensitive Przewalski's gazelle is to projected impacts of climate change across the entirety of its range. Given the constraints limited habitat space would put on gazelle population growth [33], [45], [62], the assumption of a linear relationship between abundance and range size is feasible when using Criterion A [59] to estimate the extinction rate based on projected range shifts [20] and the longer life span of gazelles [67]. Additionally, Liu *et al.* [56] suggest that even in those applications where some subjective decision making is involved, it is still useful to estimate the most appropriate thresholds while using the “objectively” determined presence/absence prediction as a reference. In this study, sensitivity analysis using different levels of threshold deduces a panorama for the extinction risk of the gazelle. This is of concern since it could reduce the arbitrary bias in assigning species to threat categories under future climate change [67]. We should acknowledge the predicted increase in extinction rate of Przewalski's gazelle under climate change despite the current degraded threat status in the IUCN Red List [35]. This could present new challenges and demands for conservation programs.

### Conservation implications

Przewalski's gazelle is one of the flagship species on the Qinghai-Tibetan Plateau, but many gazelles do not live within the protected area [36]. Moreover, the gazelle was only found in the region around Qinghai Lake where other large herbivores (e.g. *Pantholops hodgsoni*, *Equus kiang* and *Poephagus mutus* ) have experienced discernible responses to climate and environment changes [68]. In the present study, we have built models to predict the impacts of climate change and evaluate the extinction risk for the gazelle. Although projected range shifts under climate change will never be certain and cannot address the proximate causes of species extinction [3], they will be substantial for Przewalski's gazelle. Thomas *et al.* [11] suggest that any reduction in the potential range is likely to lead to an increased risk of local extinction. In this regard, the risk of extinction to Przewalski's gazelle appears to be increasing with climate change. Furthermore, if a species becomes restricted to a few sites in fragmented landscapes, just as the status for Przewalski's gazelle [33], [35], [36], local catastrophic events such as droughts or disease outbreaks or an increase of land transformation by humans could easily cause the extinction of that species [69]. It would be best to conserve all possible habitats given the endangered status of the gazelle and the uncertainty of the impacts of climate change. Efforts such as securing existing protected areas (i.e. the Qinghai Lake National Nature Reserve and the special protected zone in Gangcha County) and establishing new reserves should be undertaken in the regions projected to be suitable over longer timescales or habitats with high suitability (e.g. the north-western region close to Qinghai Lake; Fig. 3). Then migration corridors must to be established between populations of the gazelle as well as between highly suitable habitats since a large proportion of projected highly suitable habitats are under pressure from intensive human activities [33], [39], [62]. Of broader significance is that all large herbivores on the Qinghai-Tibet Plateau currently experiencing declining populations are disproportionately threatened [70]. Only combining the existing knowledge of the likely impacts of climate change, can people protect Przewalski's gazelle and other endangered large herbivores effectively.

## Supporting Information

Figure S1 **Analyzing the importance of individual predictor in the Maximum Entropy Approach (Maxent) with all selected explanatory variables.** Jackknife analyses are used to assess individual predictor importance in the development of model in relation to overall model quality or “total gain” (grid bar) at 1×1 km. Black bars indicate the gain achieved when including that predictor only and excluding remaining predictors; gray bars show how much the total gain is diminished without the given predictor. HII: human influence index; GDP: gross domestic product; bio01: annual mean temperature; bio02: mean diurnal range; bio03: isothermality; bio04: temperature seasonality; bio05/06: max/min temperature of the warmest/coldest month; bio07: temperature annual range (P5–P6); bio08/09/10/11: mean temperature of the wettest/driest/warmest/coldest quarter; bio12: annual precipitation; bio13/14: precipitation of the wettest/driest month; bio15: precipitation seasonality; bio16/17/18/19: precipitation of the wettest/driest/warmest/coldest quarter; CTI: compound topographic index; landcov: land-cover; ndvi01-12: normalized difference vegetation index (NDVI) of each month (see Hu & Jiang, 2010 for details).
(TIF)

Table S1 **Presence points of** ***Procapra przewalskii*** **based on the field work and historical records from the literature.**
(DOCX)

## References

[1] Solomon S, Qin D, Manning M, Marquis M, Averyt K (2007). Climate change 2007: the physical science basis. Contribution of Working Group I to the fourth Assessment Report of the Intergovenmental Panel on Climate Change.

[2] Root TL, Price JT, Hall KR, Schneider SH, Rosenzweig C (2003). Fingerprints of global warming on wild animals and plants.. Nature.

[3] Araújo MB, Rahbek C (2006). How does climate change affect biodiversity?. Science.

[4] Peterson AT, Ortega-Huerta MA, Bartley J, Sanchez-Cordero V, Soberon J (2002). Future projections for Mexican faunas under global climate change scenarios.. Nature.

[5] Hu J, Hu H, Jiang Z (2010). The impacts of climate change on the wintering distribution of an endangered migratory bird.. Oecologia.

[6] Singh NJ, Milner-Gulland EJ (2011). Conserving a moving target: planning protection for a migratory species as its distribution changes.. Journal of Applied Ecology.

[7] Walther GR, Post E, Convey P, Menzel A, Parmesan C (2002). Ecological responses to recent climate change.. Nature.

[8] Moritz C, Patton JL, Conroy CJ, Parra JL, White GC (2008). Impact of a century of climate change on small-mammal communities in Yosemite National Park, USA.. Science.

[9] Rosenzweig C, Karoly D, Vicarelli M, Neofotis P, Wu Q (2008). Attributing physical and biological impacts to anthropogenic climate change.. Nature.

[10] Moyes K, Nussey DH, Clements MN, Guinness FE, Morris A (2011). Advancing breeding phenology in response to environmental change in a wild red deer population.. Global Change Biology.

[11] Thomas CD, Cameron A, Green RE, Bakkenes M, Beaumont LJ (2004). Extinction risk from climate change.. Nature.

[12] Jetz W, Wilcove DS, Dobson AP (2007). Projected impacts of climate and land-use change on the global diversity of birds.. PLoS Biology.

[13] Morueta-Holme N, Fløjgaard C, Svenning JC (2010). Climate change risks and conservation implications for a threatened small-range mammal species.. PloS ONE.

[14] Ohlemüller R, Anderson BJ, Araújo MB, Butchart SHM, Kudrna O (2008). The coincidence of climatic and species rarity: high risk to small-range species from climate change.. Biology Letters.

[15] Jetz W, Rahbek C (2002). Geographic range size and determinants of avian species richness.. Science.

[16] Jansson R (2003). Global patterns in endemism explained by past climatic change.. Proceedings of the Royal Society of London Series B: Biological Sciences.

[17] Nogués-Bravo D, Rodríguez J, Hortal J, Batra P, Araújo MB (2008). Climate change, humans, and the extinction of the Woolly Mammoth.. PLoS Biology.

[18] Anderson BJ, Akçakaya HR, Araújo MB, Fordham DA, Martinez-Meyer E (2009). Dynamics of range margins for metapopulations under climate change.. Proceedings of the Royal Society B: Biological Sciences.

[19] Dobrowski SZ (2011). A climatic basis for microrefugia: the influence of terrain on climate.. Global Change Biology.

[20] Thuiller W, Broennimann O, Hughes G, Alkemade JRM, Midgley GF (2006). Vulnerability of African mammals to anthropogenic climate change under conservative land transformation assumptions.. Global Change Biology.

[21] Hijmans RJ, Graham CH (2006). The ability of climate envelope models to predict the effect of climate change on species distributions.. Global Change Biology.

[22] Parra JL, Monahan WB (2008). Variability in 20th century climate change reconstructions and its consequences for predicting geographic responses of California mammals.. Global Change Biology.

[23] Guisan A, Thuiller W (2005). Predicting species distribution: offering more than simple habitat models.. Ecology Letters.

[24] Pearson RG, Dawson TP (2003). Predicting the impacts of climate change on the distribution of species: are bioclimate envelope models useful?. Global Ecology and Biogeography.

[25] Araújo MB, Nogues-Bravo D, Diniz-Filho JAF, Haywood AM, Valdes PJ (2008). Quaternary climate changes explain diversity among reptiles and amphibians.. Ecography.

[26] Wiens JA, Stralberg D, Jongsomjit D, Howell CA, Snyder MA (2009). Niches, models, and climate change: assessing the assumptions and uncertainties.. Proceedings of the National Academy of Sciences.

[27] Araújo MB, Whittaker RJ, Ladle RJ, Erhard M (2005). Reducing uncertainty in projections of extinction risk from climate change.. Global Ecology and Biogeography.

[28] Buisson L, Thuiller W, Casajus N, Lek S, Grenouillet G (2010). Uncertainty in ensemble forecasting of species distribution.. Global Change Biology.

[29] Diniz-Filho JAF, Bini LM, Rangel TF, Loyola RD, Hof C (2009). Partitioning and mapping uncertainties in ensembles of forecasts of species turnover under climate change.. Ecography.

[30] Heikkinen RK, Luoto M, Araújo MB, Virkkala R, Thuiller W (2006). Methods and uncertainties in bioclimatic envelope modelling under climate change.. Progress in Physical Geography.

[31] Schaller G (1998). Wildlife of the Tibetan steppe.

[32] Mallon DP, Kingwood SC (2001). Antelopes. Part 4: North Africa, the Middle East, and Asia. Global Survey and Regional Action Plans.

[33] Jiang Z (2004). Przewalski's Gazelle.

[34] Jiang Z, Feng Z, Wang Z, Chen L, Cai P (1995). Historical and current distributions of Przewalski's gazelles.. Acta Theriologica Sinica.

[35] IUCN SSC Antelope Specialist Group (2008). *Procapra przewalskii*. IUCN 2010 IUCN Red List of Threatened Species Version 2010.4.. http://www.iucnredlist.org.

[36] Li C, Jiang Z, Li D, Ping X, Cai J (2011). Current status and conservation of Przewalsk's gazelle *Procapra przewalskii*.. Oryx (Accepted).

[37] Ye R, Cai P, Peng M, Lu X, Ma S (2006). The investigation about distribution and population size of Przewalski's gazelle (*Procapra przewalskii*) in Qinghai Province, China.. Acta Theriologica Sinica.

[38] Elith J, Leathwick JR (2009). Species distribution models: ecological explanation and prediction across space and time.. Annual Review of Ecology, Evolution, and Systematics.

[39] Jiang Z, Li D, Wang Z (2000). Population declines of Przewalski's gazelle around Qinghai Lake, China.. Oryx.

[40] Lei R, Jiang Z, Liu B (2001). Group pattern and social segregation in Przewalski's gazelle (*Procapra przewalskii*) around Qinghai Lake, China.. Journal of Zoology.

[41] Hijmans RJ, Cameron SE, Parra JL, Jones PG, Jarvis A (2005). Very high resolution interpolated climate surfaces for global land areas.. International Journal of Climatology.

[42] GLC (2003). Global Land Cover 2000 database. European Commission, Joint Research Centre.. http://gem.jrc.ec.europa.eu/products/glc2000/glc2000.php.

[43] Last of the Wild Data Version 2 (2005). Global Human Influence Index (HII). Wildlife Conservation (WCS) and Center for International Earth Science Information Network (CIESIN).. http://sedac.ciesin.columbia.edu/wildareas/.

[44] USGS (2009). HYDRO1k elevation derivative database.. http://eros.usgs.gov/#/Find_Data/Products_and_Data_Available/gtopo30/hydro.

[45] Hu J, Jiang Z (2010). Predicting the potential distribution of the endangered Przewalski's gazelle.. Journal of Zoology.

[46] Kim, Kim SJ, Flato, Flato G, Boer (2003). A coupled climate model simulation of the Last Glacial Maximum, Part 2: approach to equilibrium.. Climate Dynamics.

[47] Gordon HB, Farrell SP (1997). Transient climate change in the CSIRO coupled model with dynamic sea ice.. Monthly Weather Review.

[48] Collins M, Tett SFB, Cooper C (2001). The internal climate variability of HadCM3, a version of the Hadley Centre coupled model without flux adjustments.. Climate Dynamics.

[49] Phillips SJ, Anderson RP, Schapire RE (2006). Maximum entropy modeling of species geographic distributions.. Ecological Modelling.

[50] Phillips SJ, Dudík M (2008). Modeling of species distributions with Maxent: new extensions and a comprehensive evaluation.. Ecography.

[51] Elith J, Graham CH, Anderson RP, Dudík M, Ferrier S (2006). Novel methods improve prediction of species' distributions from occurrence data.. Ecography.

[52] Araújo MB, New M (2007). Ensemble forecasting of species distributions.. Trends in Ecology & Evolution.

[53] Thuiller W (2004). Patterns and uncertainties of species' range shifts under climate change.. Global Change Biology.

[54] Lawler JJ, White D, Neilson RP, Blaustein AR (2006). Predicting climate-induced range shifts: model differences and model reliability.. Global Change Biology.

[55] Marmion M, Parviainen M, Luoto M, Heikkinen RK, Thuiller W (2009). Evaluation of consensus methods in predictive species distribution modelling.. Diversity and Distributions.

[56] Liu CR, Berry PM, Dawson TP, Pearson RG (2005). Selecting thresholds of occurrence in the prediction of species distributions.. Ecography.

[57] Aranda SC, Lobo JM (2011). How well does presence-only-based species distribution modelling predict assemblage diversity? A case study of the Tenerife flora.. Ecography.

[58] Penman TD, Pike DA, Webb JK, Shine R (2010). Predicting the impact of climate change on Australia's most endangered snake, *Hoplocephalus bungaroides*.. Diversity and Distributions.

[59] IUCN (2001). IUCN Red List Categories and Criteria: Version 3.1.

[60] Klein JA, Harte J, Zhao X (2007). Experimental warming, not grazing, decreases rangeland quality on the Tibetan Plateau.. Ecological Applications.

[61] Klein J, Harte J, Zhao X (2008). Decline in medicinal and forage species with warming is mediated by plant traits on the Tibetan Plateau.. Ecosystems.

[62] Hu J, Ping X, Cai J, Li Z, Li C (2010). Do local communities support the conservation of endangered Przewalski's gazelle?. European Journal of Wildlife Research.

[63] Thuiller W, Araújo MB, Pearson RG, Whittaker RJ, Brotons L (2004). Biodiversity conservation-Uncertainty in predictions of extinction risk.. Nature.

[64] Pearson RG, Thuiller W, Araújo MB, Martinez-Meyer E, Brotons L (2006). Model-based uncertainty in species range prediction.. Journal of Biogeography.

[65] Yang J, Jiang Z, Zeng Y, Turghan M, Fang H (2011). Effect of anthropogenic landscape features on population genetic differentiation of Przewalski's gazelle: main role of human settlement.. PLoS ONE.

[66] Li C, Jiang Z, Feng Z, Yang X, Yang J (2009). Effects of highway traffic on diurnal activity of the critically endangered Przewalski's gazelle.. Wildlife Research.

[67] AkÇakaya HR, Butchart SHM, Mace GM, Stuart SN, Hilton-Taylor C (2006). Use and misuse of the IUCN Red List Criteria in projecting climate change impacts on biodiversity.. Global Change Biology.

[68] Ma R, Jiang Z (2006). Impacts of environmental degradation on wild vertebrates in the Qinghai Lake drainage.. Acta Ecologica Sinica.

[69] Lawton JH, May RM (1995). Extinction rates.

[70] Mallon DP, Jiang Z (2009). Grazers on the plains: challenges and prospects for large herbivores in Central Asia.. Journal of Applied Ecology.

