# Supplementary figures and images for: Climate Change Hastens the Conservation Urgency of an Endangered Ungulate
Source: PLoS One. 2011 Aug 3;6(8):e22873. doi: 10.1371/journal.pone.0022873 (PMC3149626; doi:10.1371/journal.pone.0022873)

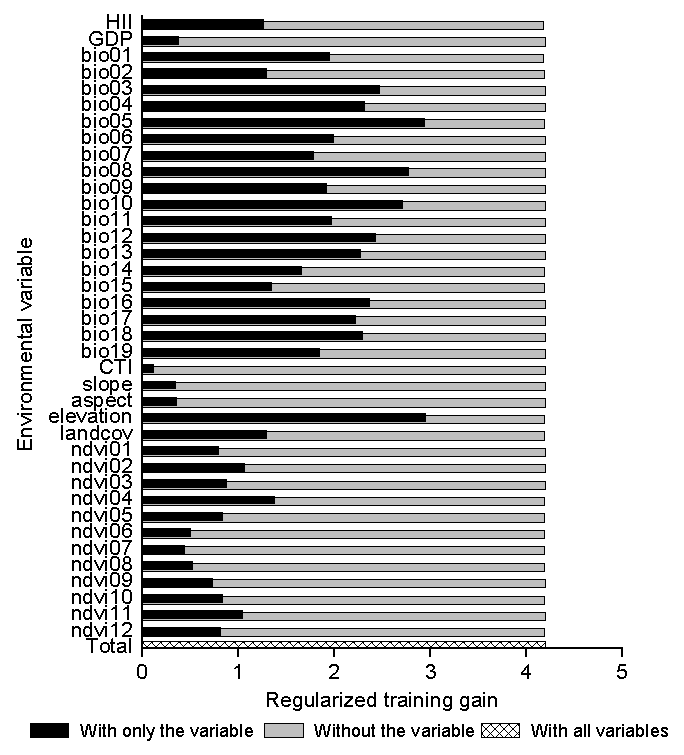

Supplement: Figure S1 — Analyzing the importance of individual predictor in the Maximum Entropy Approach (Maxent) with all selected explanatory variables. Jackknife analyses are used to assess individual predictor importance in the development of model in relation to overall model quality or “total gain” (grid bar) at 1×1 km. Black bars indicate the gain achieved when including that predictor only and excluding remaining predictors; gray bars show how much the total gain is diminished without the given predictor. HII: human influence index; GDP: gross domestic product; bio01: annual mean temperature; bio02: mean diurnal range; bio03: isothermality; bio04: temperature seasonality; bio05/06: max/min temperature of the warmest/coldest month; bio07: temperature annual range (P5–P6); bio08/09/10/11: mean temperature of the wettest/driest/warmest/coldest quarter; bio12: annual precipitation; bio13/14: precipitation of the wettest/driest month; bio15: precipitation seasonality; bio16/17/18/19: precipitation of the wettest/driest/warmest/coldest quarter; CTI: compound topographic index; landcov: land-cover; ndvi01-12: normalized difference vegetation index (NDVI) of each month (see Hu & Jiang, 2010 for details). (TIF) [file pone.0022873.s001.tif]
