# Supplementary material for: Climate Change Hastens the Conservation Urgency of an Endangered Ungulate
Source: PLoS One. 2011 Aug 3;6(8):e22873. doi: 10.1371/journal.pone.0022873 (PMC3149626; doi:10.1371/journal.pone.0022873)
Supplement: Table S1 — Presence points of Procapra przewalskii based on the field work and historical records from the literature. (DOCX) [file pone.0022873.s002.docx]

**Table S1** Presence points of *Procapra przewalskii* based on the field work and historical records from the literature.

| No. | Longitude (°) | Latitude(°) | Source | No. | Longitude (°) | Latitude(°) | Source |
| --- | --- | --- | --- | --- | --- | --- | --- |
| 1 | 96.115 | 39.327 | Jiang, 2004 | 60 | 100.467 | 37.215 | Present study |
| 2 | 98.355 | 37.427 | Present study | 61 | 100.470 | 37.218 | Present study |
| 3 | 98.359 | 37.418 | Present study | 62 | 100.478 | 37.135 | Present study |
| 4 | 98.436 | 37.500 | Present study | 63 | 100.478 | 37.165 | Present study |
| 5 | 98.440 | 37.498 | Present study | 64 | 100.480 | 37.163 | Present study |
| 6 | 98.443 | 37.492 | Present study | 65 | 100.487 | 37.101 | Present study |
| 7 | 98.478 | 37.492 | Present study | 66 | 100.493 | 37.122 | Present study |
| 8 | 98.498 | 37.472 | Present study | 67 | 100.495 | 37.094 | Present study |
| 9 | 98.498 | 37.492 | Present study | 68 | 100.497 | 37.110 | Present study |
| 10 | 98.500 | 37.471 | Present study | 69 | 100.499 | 37.115 | Present study |
| 11 | 98.503 | 37.462 | Present study | 70 | 100.499 | 37.074 | Present study |
| 12 | 98.508 | 37.458 | Present study | 71 | 100.500 | 37.104 | Present study |
| 13 | 98.518 | 37.489 | Present study | 72 | 100.503 | 37.070 | Present study |
| 14 | 98.520 | 37.454 | Present study | 73 | 100.504 | 37.112 | Present study |
| 15 | 98.521 | 37.478 | Present study | 74 | 100.507 | 37.089 | Present study |
| 16 | 98.521 | 37.517 | Present study | 75 | 100.511 | 37.109 | Present study |
| 17 | 98.523 | 37.505 | Present study | 76 | 100.513 | 37.070 | Present study |
| 18 | 98.530 | 37.457 | Present study | 77 | 100.514 | 37.096 | Present study |
| 19 | 98.538 | 37.491 | Present study | 78 | 100.515 | 37.102 | Present study |
| 20 | 98.538 | 37.470 | Present study | 79 | 100.525 | 37.096 | Present study |
| 21 | 98.538 | 37.485 | Present study | 80 | 100.531 | 37.100 | Present study |
| 22 | 98.548 | 37.459 | Present study | 81 | 100.533 | 37.082 | Present study |
| 23 | 98.555 | 37.482 | Present study | 82 | 100.702 | 36.883 | Present study |
| 24 | 98.557 | 37.475 | Present study | 83 | 100.702 | 36.869 | Present study |
| 25 | 98.559 | 37.491 | Present study | 84 | 100.712 | 36.884 | Present study |
| 26 | 98.564 | 37.481 | Present study | 85 | 100.714 | 36.890 | Present study |
| 27 | 98.565 | 37.473 | Present study | 86 | 100.716 | 36.883 | Present study |
| 28 | 98.570 | 37.451 | Present study | 87 | 100.717 | 36.872 | Present study |
| 29 | 98.573 | 37.490 | Present study | 88 | 100.729 | 36.870 | Present study |
| 30 | 98.575 | 37.492 | Present study | 89 | 100.735 | 36.865 | Present study |
| 31 | 98.576 | 37.482 | Present study | 90 | 100.742 | 36.861 | Present study |
| 32 | 98.576 | 37.471 | Present study | 91 | 100.789 | 36.584 | Present study |
| 33 | 98.582 | 37.483 | Present study | 92 | 100.796 | 36.712 | Present study |
| 34 | 98.582 | 37.491 | Present study | 93 | 100.798 | 36.812 | Present study |
| 35 | 98.593 | 37.449 | Present study | 94 | 100.798 | 36.803 | Present study |
| 36 | 98.596 | 37.482 | Present study | 95 | 100.800 | 36.804 | Present study |
| 37 | 98.601 | 37.471 | Present study | 96 | 100.801 | 36.582 | Present study |
| 38 | 98.605 | 37.479 | Present study | 97 | 100.804 | 36.811 | Present study |
| 39 | 99.259 | 37.180 | Jiang, 2004 | 98 | 100.804 | 36.710 | Present study |
| 40 | 99.312 | 36.404 | Present study | 99 | 100.811 | 36.703 | Present study |
| 41 | 99.326 | 36.394 | Present study | 100 | 100.812 | 36.567 | Present study |
| 42 | 99.881 | 36.980 | Present study | 101 | 100.814 | 36.690 | Present study |
| 43 | 99.886 | 36.976 | Present study | 102 | 100.816 | 36.699 | Present study |
| 44 | 99.886 | 37.010 | Present study | 103 | 100.821 | 36.812 | Present study |
| 45 | 99.890 | 36.985 | Present study | 104 | 100.823 | 36.695 | Present study |
| 46 | 99.899 | 37.005 | Present study | 105 | 100.823 | 36.709 | Present study |
| 47 | 100.431 | 37.192 | Present study | 106 | 100.825 | 36.697 | Present study |
| 48 | 100.440 | 37.191 | Present study | 107 | 100.827 | 36.685 | Present study |
| 49 | 100.443 | 37.193 | Present study | 108 | 100.829 | 36.679 | Present study |
| 50 | 100.448 | 37.155 | Present study | 109 | 100.830 | 36.825 | Present study |
| 51 | 100.449 | 37.184 | Present study | 110 | 100.831 | 36.818 | Present study |
| 52 | 100.451 | 37.161 | Present study | 111 | 100.836 | 36.819 | Present study |
| 53 | 100.456 | 37.191 | Present study | 112 | 100.842 | 36.827 | Present study |
| 54 | 100.457 | 37.155 | Present study | 113 | 101.646 | 37.843 | Jiang, 2004 |
| 55 | 100.460 | 37.174 | Present study | 114 | 101.693 | 37.857 | Jiang, 2004 |
| 56 | 100.461 | 37.152 | Present study | 115 | 106.132 | 38.518 | Jiang, 2004 |
| 57 | 100.465 | 37.161 | Present study | 116 | 107.597 | 38.279 | Jiang, 2004 |
| 58 | 100.466 | 37.186 | Present study | 117 | 109.917 | 40.639 | Jiang, 2004 |
| 59 | 100.467 | 37.148 | Present study |  |  |  |  |
